# *Megalara garuda*, a new genus and species of larrine wasps from Indonesia (Larrinae, Crabronidae, Hymenoptera)

**DOI:** 10.3897/zookeys.177.2475

**Published:** 2012-03-23

**Authors:** Lynn S. Kimsey, Michael Ohl

**Affiliations:** 1 Bohart Museum of Entomology, University of California, Davis, 95616, USA; 2 Museum fuer Naturkunde, Leibniz-Institut fuer Evolutions und Biodiversitaetsforschung an der Humboldt-Universität zu Berlin, Germany

**Keywords:** Sulawesi

## Abstract

A new genus and species, *Megalara garuda* is described from Sulawesi (Indonesia). The new species is one the largest known members of the crabronid subfamily Larrinae. It has a unique suite of putatively apomorphic morphological characters and is most closely related to the genus *Paraliris*. We found indications of a significant allometric variation in body size and mandibular length and shape in male *Megalara*, and the presence of acarinaria at least in females of the new genus. Allometric variation and acarinaria have previously been shown to occur in *Paraliris*, which is another indication for a close relationship of *Megalara* and *Paraliris*.

## Introduction

A survey of the Mekongga Mountains and Papalia west and south of Kendari in southeastern Sulawesi uncovered a spectacular new genus and species of larrine crabronid. This new species was also independently discovered among unidentified, historical apoid wasps in the Museum fuer naturkunde in Berlin. It appears to be most closely related to the Southeast Asian genera *Dalara* and *Paraliris*. *Dalara* contains two species, *mandibularis* (F. X. Williams) from the Philippines and *schlegelii* (Ritsema) from Sumatra and *Paraliris* has four currently known species, *faceta* (Bingham) from India and Burma, *kriechbaumeri* Kohl from Java, *sycorax* (F. Smith) and *truncatus* van der Vecht, both from Borneo.

This new species from Sulawesi is among the largest members of the Larrini, with the majority of males greater than 3 cm in length. Only some *Liris* are as long as 30 mm (Menke in Bohart and Menke 1976). Two male morphs were discovered. One about the size of the females and the other much larger with exaggerated male features, such as the length and tooth development of the mandibles, flattening of the face and prolongation of the gena and vertex. The biology of this new genus is unknown.

## Materials and methods

Mekongga and Papalia specimens were collected in Townes-style Malaise traps. Specimen images were taken with a Nikon D300 digital camera and assembled using the CombineZM software™. The holotype is deposited in the Museum Zoologicum Bogorense, Chibinong, Indonesia. Paratypes are deposited in the Museum fuer Naturkunde, Berlin, Germany, the Bohart Museum of Entomology, University of California, Davis, USA; Museum Zoologicum Bogorense, and the National Museum of Natural History (Naturalis), Leiden, the Netherlands.

### Megalara

Kimsey & Ohl gen. n.

urn:lsid:zoobank.org:act:8107BDD7-6A10-4658-AD75-2A74646C9D47

http://species-id.net/wiki/Megalara

#### Type species.

*Megalara garuda* Kimsey and Ohl, new species.

#### Generic diagnosis.

*Megalara* is closely related to *Liris* Fabricius, *Larra* Fabricius, *Dalara*, *Paraliris*, and *Dicranorhina* Shuckard, which together are classified as Larrina (Pulawski 2011) based on the presence of a horizontal swelling below the midocellus combined with a vertical swelling along the inner eye margin. The new genus is similar to *Dalara* and *Paraliris* in having the mandibles bidentate apically or very long, at most with a weak notch on outer margin. Within this group of genera, *Megalara* can be distinguished by the large body size (25-34 mm; 20-24 mm in *Paraliris*). A unique character of *Megalara* is the markedly developed malar space, whereas all other genera in the Larrina have the malar space narrow or virtually absent. Additionally, the propodeal dorsum is punctate in *Megalara*, but finely, transversely striatorugose in *Dalara* and *Paraliris*. A subsidiary recognition character is the dark body pubescence, which is markedly developed in females, but indistinct in males (body covered with dense, silvery pubescence in *Paraliris*, erect pubescence lacking at all in *Dalara*).

Males of *Megalara* have a number of complementary diagnostic characters: One of the most remarkable characters is the enormously enlarged male mandibles, which are almost as long as the forelegs. All species of *Dalara* and some of *Paraliris* also have elongate mandibles in the males, but shape and dentition are different. In *Megalara*, the mandibular apex is simple and the inner, subbasal tooth is greatly enlarged. In *Dalara*, the male mandible has one small subapical and one large subbasal tooth, whereas in *Paraliris*, the mandible is broadly bidentate apically in minor males and lacks subapical and subbasal teeth in major males with elongate mandibles. Other male characters diagnostic for *Megalara* are the hindfemoral venter, which is longitudinally compressed (evenly, slightly convex in *Paraliris*, with a hook-like process or prominent bulge toward base in *Dalara*), metasomal sterna III-IV with paired submedial lobes (simple in *Paraliris* and *Dalara*), sternum VIII bilobed apically (truncate in *Paraliris*, roundly truncate with a small median notch in *Dalara*), and the penis valve with a longitudinal row of markedly strong teeth on the ventral side (in *Paraliris*, penis valve with inwards directed apical and medial process, without ventral teeth).

Females of *Megalara* and *Paraliris* are generally quite similar and can be distinguished by the larger body size of *Megalara*, the dark pubescence in *Megalara* (silvery in *Paraliris*), and the propodeal dorsum, which is punctate in *Megalara* and finely, transversely striatorugose in *Paraliris*. Female *Megalara* and *Dalara* can be readily differentiated by dull or weakly, shining virtually asetose metasomal terga and the significantly different body sizes.

#### Partial modification of the key to Old World genera of Larrini by Bohart and Menke (1976), replacing couplet 6

**Table:** 

6	Metasomal terga dull or weakly shining, impunctate or evenly, sparsely punctate. Body without erect pilosity, at most with thin pruinose pubescence. Wings hyaline except for cloudy areas at marginal and costal cells. Clypeus with truncate lobe, which is narrower than outer distance of antennal sockets. Male hindfemur with hook-like process or prominent bulge toward base. Body length not exceeding 12 mm.	*Dalara* Ritsema
–	Metasomal terga shining, lateral portions of at least terga II-V coarsely punctatorugose, tergal disks impuncate or sparsely punctate. Body completely or partly covered with erect pilosity. Wings infumate. Clypeus with truncate or broadly emarginate lobe, which is significantly broader than outer distance of antennal sockets. Male hindfemur unmodified or longitudinally compressed below. Body length 17 to 34 mm.	6a
6a	Body covered with dense, silvery pubescence. Propodeal dorsum finely, transversely striatorugose. Malar space narrow to virtually absent. Mandibular apex broadly bidentate in both sexes, if mandibles unusually long and sickle-shaped (major males), then apex simple and submedial tooth or inner margin lacking. Male: forefemoral venter with long, dense pubescence; hindfemur and sterna simple; apex of sternum VIII truncate; penis valve with inwards directed apical and medial process. Male body length 20–24 mm.	*Paraliris* Kohl
–	Body covered with dark pubescence, well-developed in females and indistinct in males. Propodeal dorsum punctate, punctures markedly dense along midline. Malar space markedly large, about 1.5–2.0 × inner distance of antennal sockets. Female mandibular apex broadly bidentate, male mandible unusually elongate, apex simple, with large submedial tooth on inner margin. Male: forefemoral venter virtually asetose; hindfemoral venter longitudinally compressed; sterna III–IV with paired submedial lobes; apex of sternum VIII with two large, finger-like processes; penis valve with longitudinal row of strong teeth ventrally. Male body length 25–34 mm	*Megalara* Kimsey & Ohl, gen. n.

#### Discussion.

*Dalara*, *Paraliris*, and *Megalara*, the newly described genus, share many morphological details with *Liris*. Menke in Bohart and Menke (1976) said that *Dalara* ‘possibly should be considered as a subgenus of *Liris* … although the biology argues otherwise’. *Liris* is a morphologically diverse genus and has been split up into several genera or subgenera by various authors in the past (see Bohart and Menke 1976, for a taxonomic summary). It includes more than 310 currently valid species and its monophyly has never been formally tested. The newly described species, *Megalara garuda*, shares a number of significant characters with *Paraliris*, and we have considered placing *Megalara garuda* as an aberrant species in this genus. However, despite the similarities, the morphological differences are so striking, that we feel that the systematic position quite apart from all other species in *Paraliris* is best reflected by placing it in a genus of its own. Thus despite the lack of a comprehensive phylogenetic analysis of the genera in the Larrini, *Dalara*, *Paraliris*, and *Megalara* each exhibit a unique set of putatively apomorphic characters, so that the taxonomic status of all of them as distinct genera seems to be fully justified. However, a formal phylogenetic analysis of the Larrini on the genus level is desperately needed to test this assumption.

The enormously enlarged male mandibles of *Megalara* are quite similar to that of male *Dalara*, but *Megalara* is more similar to *Paraliris* in most details. *Paraliris* has been revised by van der Vecht (1982), and one of us (MO) reexamined most of the specimens of the genus, which van der Vecht studied. Van der Vecht pointed out that males of at least *Paraliris kriechbaumeri*, show a remarkable amount of allometric variation with respect to general body size and mandibular length. Male body length varies from 15 to 22 mm, and the mandibles of the smallest males are very similar to female mandibles in being stout and broadly bidentate apically, whereas the mandibles of the largest males are elongate, sickle-shaped and simply apically and basally (van der Vecht 1982: Figs 2–4). Despite these enormous differences particularly in mandibular size and shape, van der Vecht felt confident, based on a study by de Beaumont (1943), that this represents an unusually broad range of intraspecific variation.

This hypothesis seems to be well founded, and we observe the same phenomenon in the newly described *Megalara*. There are two male morphs with remarkable differences in body size and mandibular size. Minor males, which have relatively short, female-like mandibles, are 25 mm long, whereas major males with exaggerated mandibles have a body length of 32 to 34 mm. However, the genital foramen and genital capsule of the minor male was packed with mites and it may also be that this individual was feminized by the heavy mite load. Unfortunately, the genital capsule was lost.

Van der Vecht (1982) also pointed out that *Paraliris* bears ‘acarinaria’, which are specialized, typically pouch-like structures in aculeate Hymenoptera, which function to carry phoretic mites. In *Paraliris*, acarinaria are located beneath a lamella near the base of terga II–V (females) and II–VI (males). These areas are dorsally covered by the overhanging posterior margin of the tergum before the acarinaria-carrying tergum. Therefore, if present, phoretic mites in acarinaria can be seen in the intersegmental gap beneath the tergal overhang even in complete specimens. In *Megalara*, we observed mites in both sexes as follows:

Males. 1 major male: 2 mites between terga II and III

Minor male: genitalia packed with mites

Females. 1 female: 10 mites between terga II and III

1 female: 16 mites between terga II and III, 6 between III and IV

The presence of two mites on the terga and the large number in the genitalia in males are probably no indication of acarinaria in *Megalara*. However, the large number of mites in the females indicates that *Megalara* also possesses mite-bearing structures. Due to the small number of specimens of the new species, we refrained from dissecting the type specimens to verify the presence of acarinaria. Presence of acarinaria in *Paraliris* and putatively in *Megalara* also is another indication of the close relationships between these genera.

#### Etymology.

The new genus name is an arbitrary combination of *Mega*-, deriving from the Greek megas, meaning large and mighty, and –*lara*, the last syllable of *Dalara*. It is an allusion both to the exaggerated body size of the new genus and its overall similarity to *Dalara*.

### Megalara garuda

Kimsey & Ohl sp. n.

urn:lsid:zoobank.org:act:60D11831-50F8-4A27-923E-8B85298DDF6F

http://species-id.net/wiki/Megalara_garuda

Figs 1 [Fig F2] 8

#### Diagnosis.

As for the genus (*vide supra*).

#### Description.

**Figures 1, 2.**
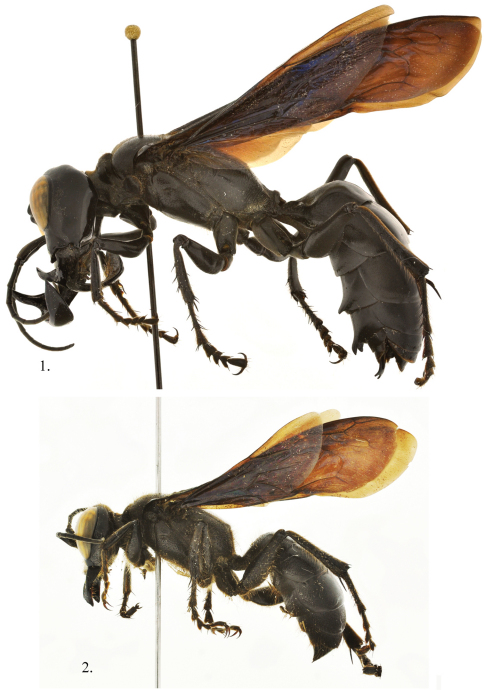
*Megalara garuda* side view of body **1** male **2** female.

**Figures 3, 4. F2:**
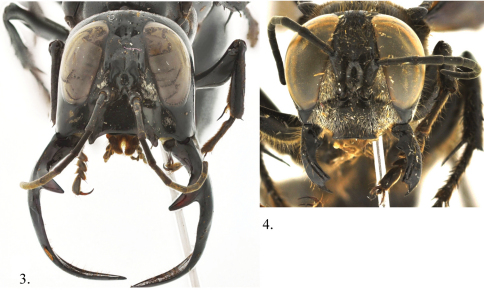
*Megalara garuda* front view of face **3** male **4** female.

**Male (major)**. Body length 32–34 mm (side view, Fig. 1). Forewing 24–25 mm. Head (Fig. 3): face strongly concave at antennal sockets; clypeus 3.5× as broad as long, flattened medially with sublateral tooth on apical margin, concave between; frons bulging with strong longitudinal swelling on either side of midocellus; hindocelli tiny circular; vertex swollen above hindocelli with medial longitudinal groove terminating in deep pit; gena expanded, wider than eye in lateral view; mandibles enlarged and elongate, apex reaching lower one-third of eye when closed, subsidiary tooth subbasal and projecting anteriorly, mandible widest subapically, longer than head in front view; occiput deeply concave; genal bridge forming apical lobe; antenna slender, flagellomere I 4× as long as broad, flagellomere II 3.6× as long as broad, flagellomere III 3.4× as long as broad, flagellomere XI 3.5× as long as broad; with weak placoids on flagellomeres III–XI; head shiny, nearly impunctate, punctures tiny, scattered; mandible highly polished; wing apices finely plicate.

Mesosoma: pronotum, scutum, scutellum, mesopleuron with scattered small punctures, 1–2 PD apart; metanotal punctures 0.5–1.0 PD apart; propodeum with medial longitudinal carina joining carina along anterior margin, dorsomedially finely transversely rugose, with dense nearly contiguous punctures, laterally becoming increasingly sparsely punctate, posterior surface marked by small dorsomedial projection, posteriorly densely transversely cross-ridged, ridges becoming smaller and finer medially; hindfemur widened ventrally in basal half, longitudinally compressed; hindtibia with crenulate ventral ridge.

**Figures 5–8.**
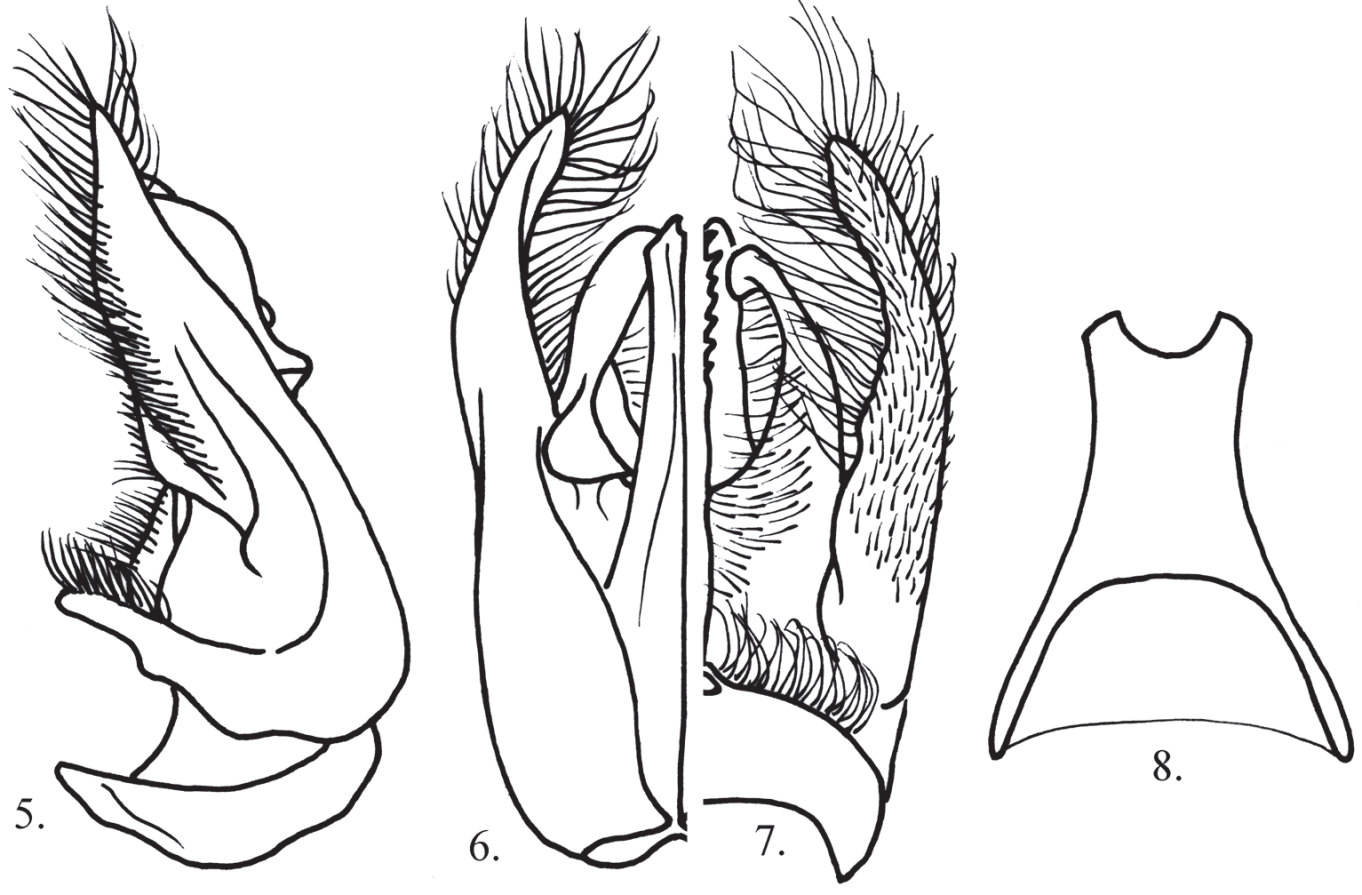
*Megalara garuda* male genital capsule **5** lateral view **6** dorsal view **7** ventral view **8** male sternum VIII.

Metasoma: polished, sparsely punctate, punctures tiny, 2–4 PD apart; tergum I with short sublateral ridge extending posteriorly from base, mediad of spiracle; terga I and II with lateral arcuate carina delimiting lateral rugosopunctate area; sternum I finely, densely rugose, with blade-like medial ridge ending in apicolateral carina delimiting short triangular posterior declivity; sterna II–III with subbasal ovoid, mat, often discolored patch (discolored to reddish in some individuals); sterna III–IV with strongly projecting sublateral, digitate lobe; sternum VIII elongate, parallel-sided apically, apex bidentate (Fig. 8). Genital capsule (Fig. 5–6).

**Male (minor)** (Features that differ from major males). Body length 25 mm. Forewing 21 mm. Mandible shorter than head in front view; clypeus 2.7× as long as broad; flagellomere I 3.6× as long as broad; flagellomere II 3.2× as long as broad; flagellomere III 2.8× as long as broad; flagellomere XI 3× as long as broad.

**Female** (Features that differ from males). Body length 20–22 mm (side view, Fig. 2); forewing 19–20 mm long. Head (Fig. 4): median lobe of clypeus truncate, broad, laterally angulate; mandible with subbasal tooth, apically bidentate; head punctures tiny, 2–3 PD apart. Mesosoma: female mesosoma including legs covered by rather dense, long, brownish pubescence; pronotal punctures 1–2 PD apart; scutum, scutellum, Metanotum 0.5–1.0 PD apart becoming denser laterally; propodeum dorsally and posteriorly densely rugospunctate less dense laterally; hindfemur without ventral carina. Metasoma: sternum I with strong median carina; sternum I–-III each with pair of oval, shiny areas, very large on II, small on III; tergum I punctures 0.5–12.0 PD apart, with impunctate band along posterior margin; terga II-III punctures 1–4 PD apart, with impunctate band along posterior margin; terga IV-V with large punctures interspersed between dense contiguous tiny punctures; tergum VI with V-shaped, carina margined pygidium, punctures longitudinally striatiform, contiguous to 1 PD apart.

#### Etymology.

Because of the spectacular appearance of the major male of this species, it is named after the “Garuda”, the national symbol of Indonesia; a mythical bird-like, warrior creature.

#### Type material.

Holotype male; Indonesia: se Sulawesi, North Kolaka, Wawo, Tinukari, S03°38'08", E121°04'34", 198 m, 23 Dec. 2009, Nugroho, Ubaidillah, Darmawan & Giyanto colrs., cacao plantation. Paratypes: Indonesia: Sulawesi: 1 male, 1 female: Kendari, South Konawe, Moramo, Sumber sari, S04°13'32", E122°44'09", 75 m, 17–31 Dec. 2009, Nugroho, Ubaidillah, Darmawan & Giyanto colrs., Malaise trap; 2 females: 31 Dec. 2009, Malaise trap (Museum Bogorense, Bohart Museum); 2 males: Ila-Ila, 500–800m, early Dec 1930, G. Heinrich (Museum für Naturkunde); 1 female: se Sulawesi, nr Sanggona, Base Camp, Gn. Watuwila, Malaise trap, c. 200m, 12–15 Oct 1989, C. van Achterberg (Leiden Museum).

## Supplementary Material

XML Treatment for Megalara

XML Treatment for Megalara garuda
